# Knowledge, attitudes, and practices of Female Genital Mutilation / Cutting among healthcare providers in two public hospitals in Egypt: A cross-sectional study

**DOI:** 10.1371/journal.pgph.0002724

**Published:** 2023-12-29

**Authors:** Ahmed Hamdy, Ahmed Taha Aboushady, Hatem Ibrahim Abd El Moty, Mohammed Omar Mostafa ELShobary, Yasmin Bassiouny, Amira Aly Hegazy

**Affiliations:** 1 Faculty of Medicine, Department of Obstetrics and Gynecology, Cairo University, Cairo, Egypt; 2 Faculty of Medicine, Alexandria University, Alexandria, Egypt; 3 Department of Obstetrics and Gynecology at Ministry of Health Al- Menshawi General Hospital, Tanta, Egypt; 4 Faculty of Medicine, Department of Community Medicine and Public Health, Cairo University, Cairo, Egypt; PLOS: Public Library of Science, UNITED STATES

## Abstract

Female Genital Mutilation / Cutting (FGM/C), also known as female circumcision, is a human rights violation and is still happening to date. Every woman or girl has the right to be protected from this harmful practice. Egypt has adopted a multi-layered strategy to end FGM/C nationwide. Even though considerable progress has been made throughout the country, the practice and inequality still exist. In 2021, The Egyptian Family Health Survey results showed a decrease in the prevalence of circumcision among ever-married women, reaching about 86%, compared to 92% in the latest public estimate of the Demographic Health Survey 2015, where 87% of all women between 15 and 49 years old are circumcised, of which 42.4% reported being circumcised by a healthcare professional (HCP) compared to a reported 47% in 2021. This study aimed to assess healthcare providers’ knowledge, attitudes, and practices in two public hospitals in 2 governorates in Egypt using a validated questionnaire conducted among HCPs in Cairo (Urban) and Gharbia (Rural) governorates. A pre-tested questionnaire comprising 38 close-ended questions was used. The study population included 223 HCPs in Cairo and Gharbia governorates, of which 63.7% were women and 36.3% were men, with an average age of 42 years (42±5). 49.8% of the respondents are from an urban area. In the knowledge domain, the highest consequence identified was reduced sexual feelings. In attitudes, almost 63% believed that FGM/C should continue, while 65% agreed that the HCPs have a role in eliminating FGM/C. Almost 4% of our respondents have performed an FGM before, 45% had FGM in their household, and 62% would perform FGM on their daughters. HCPs’ integration within the communities allows them to play a crucial role in preventing the practice. It is of utmost importance to compensate for the gap in the curricula of medical schools through informal learning activities and continuing medical education programs for sexual and reproductive health and rights and human rights, as legislation and law enforcement alone cannot eliminate FGM/C from society.

## Introduction

Female Genital Mutilation / Cutting (FGM/C), also known as female circumcision, is a human rights violation and is still happening to date. It is a manifestation of entrenched gender inequality with catastrophic and life-long consequences. Women and girls have the right to be protected from this harmful practice. FGM/C is now part of the Sustainable Development Agenda through its inclusion in the Sustainable Development Goal (SDG) target 5.3, aiming to eliminate the practice by 2030 [1]. The 2008 UN interagency statement condemns FGM/C, as it violates several human rights conventions, including the Universal Declaration of Human Rights, the Convention on the Elimination of All Forms of Discrimination against Women (CEDAW), and the Convention on the Rights of the Child [2]. According to estimates, 200 million women worldwide are mutilated, and more than 3 million girls are at risk yearly [3, 4].

FGM/C covers practices that purposefully damage or change female genitalia for non-medical reasons. FGM/C is a harmful traditional practice carried out on young girls under 15, typically performed by a traditional circumciser (most commonly Dayas) using a blade in unsafe circumstances, fueled by numerous societal norms and cultural beliefs [5, 6]. Having medical personnel perform the process to make it safer is opposed because it is widely acknowledged that doing so violates the human rights of women and girls [7].

The World Health Organization (WHO) defines medicalization as "the situation in which any category of healthcare provider practices FGM/C, whether in a public or a private clinic, at home or elsewhere" These HCPs include physicians, nurses, and/or midwives [8]. Data from the Demographic and Health Surveys (DHS) in several countries show that medicalization has increased in Egypt, Guinea, Indonesia, Kenya, Nigeria, Sudan, and Yemen. In several countries, at least one-third of the women respondents reported that a trained healthcare provider mutilated their daughters [9–11].

The practice is prevalent in 30 countries in Africa’s west, east, and northeast parts, various Middle Eastern and Asian countries, and among immigrants from these regions [12]. Certain ethnic groups in Central and South America have also reported this practice. The practice is most reported in Somalia (98%), Guinea (97%), and Djibouti (93%) among women and girls aged 15–49 [13]. FGM/C has been criminalized or restricted in most nations where it occurs, and there have been worldwide efforts to convince practitioners to stop doing it [12]. The rising of international migration and mobility has increased the number of women and girls in Europe, the United States, Australia, and Canada who have or are at risk of this practice, making this issue more widely acknowledged in nations where FGM/C is not usually performed [14] and thus, making it a violation of international concern [15]. The European Institute of Gender Equality estimates that in Europe, 180,000 women and girls are at risk for FGM/C annually [16–18].

Even though some progress has been made throughout the country in some aspects of FGM practices, inequality exists. In some governorates, the practice remains universal and far from the SDG targets to eliminate FGM. To achieve elimination by 2030, progress needs to be 15 times faster, acknowledging the observed decline in the last 15 years. According to WHO’s classification of FGM/C into four types [19], in Egypt, the most common types are; types I (clitoridectomy) and II (clitoridectomy + (partial) removal of the labia minora) [20]. In contrast, type III (infibulations) is rare, as is type IV (other forms), and the practice is usually performed before puberty [5], with a median age of 9 years [19]. All types of FGM can cause adverse consequences that significantly threaten the health and welfare of newborns, girls, and women, demanding urgent attention within the global sexual and reproductive health and rights agenda. The immediate aftermath of FGM can result in shock, bleeding, and profound psychological consequences, further compounded by infections when performed by unhygienic or untrained individuals. Moreover, the long-term consequences may include chronic pain, keloids, fibrosis, heightened childbirth complications, primary infertility, and enduring psychological trauma [21, 22].

The justifications for FGM/C differ from region to region. It is a crucial aspect of raising a girl and a technique to prepare her for marriage and adulthood. Ideas about proper and acceptable sexual conduct frequently drive it. FGM/C is considered a social convention (social norm), accompanied by social pressure to conform to societal norms, a need to fit in, and a fear of rejection by the community [22]. Despite the declining rates of FGM/C, with the fast population growth in many of the practicing countries, more women and girls will be at risk, and this will likely increase by 2030 [13, 23].

In Egypt, the Demographic Health Survey (EDHS) of 2015 showed 87% of previously married women between 15 and 49 years old were circumcised, vs. 86% in the 2022 Egyptian Family Health Survey (EFHS), of which 42.4% reported being circumcised by a healthcare provider (HCP) in the 2015 survey vs. 47% in the 2021 EFHS survey. Girls under 15 years old are four times more likely than women between 40 to 45 years to have been mutilated by a healthcare worker [24–26].

FGM/C medicalization is still surging dramatically in Egypt. Egypt has the most significant rate of medicalized FGM/C among the nations that practice the operation [27]. The campaigns to end FGM/C, where much of the messaging emphasized the immediate physical effects of FGM/C prompting mothers to choose for their daughters to be cut by HCPs, may be somewhat to blame for this increase. Regarding their attitude, only 26% of men and 37.5% of women are against continuing this practice in Egypt and believe it should be stopped, compared to 53.9% of the women and 52.6% of the men who believe that FGM/C should continue. Moreover, 46.2% believe it’s required by religion [27].

Egypt has adopted a multi-layered strategy to end FGM/C nationwide, starting with the Ministry of Health and Population’s 1997 medical decree, which restricted FGM/C only to be carried out by doctors at predetermined facilities, which may also be responsible for this surge [28]. The original purpose of this ruling was to lessen complications and ultimately put an end to the practice. However, the subsequent deaths who underwent mutilation in hospitals pressured the ministry to amend the decree in 2007 and ban the practice in all hospitals [29]. Later in 2016, the Parliament subsequently issued the law with increased prison terms for offenders between 5 and 7 years and harsher sentences, up to 15 years, if the procedure leads to death or deformity [30]. Furthermore, in May 2018, the Egyptian Dar Al-Iftaa (Centre for Islamic Legal Research) ruled significantly and supported the FGM/C ban. They announced that FGM/C is religiously forbidden and that Islamic laws do not require the practice [31]. Additionally, despite scarce evidence on the Christian views on FGM, all Christian authorities unanimously agree that FGM I not supported in the religious texts [32]. While religious law does not prescribe FGM, certain practitioners may perceive it as a religious obligation due to the importance of female sexual purity, which holds significance across all monotheistic religions [33].

Despite the government’s effort, supported by many stakeholders, to combat FGM/C, the practice is still stubbornly persistent. Although several studies investigated the Knowledge and attitudes, and practices of healthcare providers in Egypt, few recent articles compared health facilities in urban and rural areas and included HCPs who may commit or be pressured to commit such practice.

This study assesses healthcare providers (HCPs) knowledge, attitudes, and practices in two public hospitals in 2 governorates in Egypt and their underlying demographic characteristics. It is shedding light on the HCP’s beliefs towards FGM, identifying the underlying factors contributing to such a public health problem, and the medicalization of such human rights violations and traditional practice. Through this study, we aim to investigate the adequacy of the HCPs’ knowledge about FGM/C, their rationale to support such practice, their attitudes towards the practice and its medicalization, and the underlying demographic characteristics.

## Methods

This bi-centric cross-sectional descriptive study aimed at analyzing data related to HCPs’ perceptions regarding FGM/C, divided into knowledge, attitude, and practices (KAP) categories. The study was conducted in two large public tertiary hospitals, namely Kasr Alainy University Hospital in Cairo, an urban governorate, and Almenshawi general hospital in Gharbia, a rural governorate.

Based on previous studies and Egypt’s DHS, the authors hypothesized that rural and female participants would be more supportive of FGM [24, 33]. Furthermore, as per the national religious institutions’ unified stance, the authors hypothesized that FGM is a cultural habit rather than a religious habit, thus not affected by religion [32].

223 HCPs in Cairo and Gharbia governorates from both genders were included, staff members including doctors and nurses at both Cairo University Hospitals (n = 111) and Al Menshawi General Hospital (n = 112), all HCPs were informed about the study and were included in the study after their approval, they expressed a high interest to collaborate in this crucial topic in Egypt. The implementation was through a self-administered written KAP questionnaire collected data.

The questionnaire included 4 sections; (1) Knowledge of FGM/C consequences, all are valid potential consequences reported by different cases; (2) Attitudes and (3) Practice, which both reflect the attitudes and practices of the HCPs; finally, (4) reasons to support, all of which are invalid reasons, as FGM has been condemned by the religious, healthcare and national authorities.

The questionnaire included 38 questions to collect socio-demographic data and information regarding the KAP of HCPs. The study questionnaire was adopted and modified from the questionnaire formulated by Marcusan AK and her colleagues to test the knowledge, attitudes, and practices of female genital mutilation/cutting among healthcare professionals in Gambia in 2013 (https://pubmed.ncbi.nlm.nih.gov/24040762/). The questionnaire was designed in collaboration with the community medicine department at Kasr Alainy Cairo University; the questionnaire was translated into Arabic, then back-translated and piloted among 10 HCPs in a different public hospital to fit the Egyptian context. The English questionnaire is included in S1 Questionnaire.

The sample size was based on evidence from previous similar studies (Marcusan et al., 2016) and (EDHS, 2014) and considered the proportion of HCWs who think that practice of FGM should be continued as a primary outcome. Epi-calc 2000 [34] was used to calculate the sample size of this cross-sectional analytical study. Assuming 80% power, 0.05 level of significance, an estimated proportion of 43% and 53% null hypothesis value, and a sample size of 194 participants was calculated, then considering the drop-out rate of 15%, the final sample size was 223 participants. In this study, we used a purposive sampling approach.

Data was first entered using Microsoft Office Excel Software Program, and the collected data was cleaned and revised for completeness and logical consistency. The data was then coded and analyzed using the Statistical Package of Social Science Software program, version 26 (SPSS (Statistical Package of Social Science)). Simple frequencies and percentages for qualitative variables mean & standard deviation for normally distributed quantitative variables, and median & quartiles for quantitative variables which are not normally distributed. The association between different variables was tested using the Chi-square test, where the p-value was significant if less than 0.05.

### Ethical approval and consent to participate

The study was approved by the research ethics committee of Kasr Alainy Faculty of Medicine, Cairo University (Approval no.: MS-101-2021). In addition to the approval of the hospital leadership to enable the dissemination of the survey amongst the hospital’s staff. All procedures for data collection were treated with confidentiality according to the Helsinki Declaration of biomedical ethics [35]. Given the sensitive nature of the questions, respondents were allowed to skip any question they were uncomfortable answering, and their confidentiality and anonymity were guaranteed by identifying them by codes. No personal identifiers were collected.

Informed written consent was obtained from each respondent detailing the impact & the objectives of the study and the confidentiality of the collected data: "Your participation in this survey is completely voluntary. All the information you provide for the study will be kept completely confidential. We record your responses, but the questionnaire will not have your name on it, and your responses to our questions are identified only by a number, never by name. We hope that this survey will be considered a baseline assessment that will guide us in improving the knowledge and attitude of HCPs towards FGM/C, which is considered an important step in combating it. The survey will take about 10–15 minutes." All participants agreed and provided informed consent before participating.

## Results

The study population included 223 HCPs in Cairo and Gharbia governorates from both genders. Staff members, including (doctors and nurses) at both Cairo University Hospitals and Al Menshawi General Hospital. Table 1 shows the breakdown profile of the respondents, consisting of 223 HCPs (63.7% female and 36.3% men), with an average age of 42 years (42±5). 49.8% of the respondents are from an urban area. Most of the participants had a master’s degree. Twenty-nine (13%) working in obstetrics and gynecology, 35 (15.7%) in anesthesiology, 39 (17.5%) in emergency, 32 (14.3%) in intensive care, 28 (12.6%) in internal medicine, 32 (14%) in physiotherapy/rehabilitation, and 28 (12.6%) in surgery.

**Table 1:** Socio-demographic characteristics.

		N	%
**Mean age, years**		223	Mean 42 ± 5
**Sex**	Male	81	36.3%
	Female	142	63.7%
**Residence**	Urban (Cairo)	111	49.8%
	Rural (Gharbia)	112	50.2%
**Religion**	Muslim	190	85.2%
	Christian	33	14.8%
**Duration of working in this field**	Less than 1 year	34	15.3%
	1 to 5 years	44	19.7%
	6 to 10 years	26	11.7%
	11 to 15 years	31	13.9%
	16 to 20 years	47	21.1%
	21 years or more	41	18.4%
**Education level**	Bachelor’s degree (MBBCh)	61	27.4%
	Diploma	8	3.6%
	Fellowship	3	1.3%
	Master’s degree	89	39.9%
	PH.D./MD	62	27.8%

*N = number of respondents

% = Percent from total participants

Table 2 presents the aggregated results of the response. The tables also include the total number of responses for each question. In the knowledge domain, the highest consequence identified was reduced sexual feelings. In attitudes, almost 63% believed that FGM/C should continue, while 65% agreed that the HCPs have a role in eliminating FGM/C. Almost 4% of our respondents have performed an FGM before, 45% had FGM in their household, and 62% would perform FGM on their daughters. The exact aggregated values in the below table and the breakdown by select socio-demographic variables (place of residence, gender, religion) can be found in Tables 3–5.

**Table 2:** The overall results of the responses.

	Yes		No		Total
**Knowledge of FGM/C consequences:**	n	%	n	%	n
**Health problems**	90	40.36	133	59.64	223
**Bleeding**	90	40.36	133	59.64	223
**Transmission of infectious diseases**	90	40.36	133	59.64	223
**Difficulty during delivery**	92	41.26	131	58.74	223
**Reduction of sexual feelings**	169	75.78	54	24.22	223
**It affects the health and welfare of women and girls**	88	39.46	135	60.54	223
**Difficult penetration during sex**	87	39.01	136	60.99	223
**No consequences**	50	22.42	173	77.58	223
**Has seen a girl with complications after FGM/C**	91	40.81	132	59.19	223
**Attitudes**	n	%	n	%	n
**Do you think that the practice of FGM/C should continue?**	141	63.23	82	36.77	223
**Do you think girls who have not undergone FGM/C should be discriminated against?**	80	35.87	143	64.13	223
**Do you think the practice of FGM/C can ever be eliminated in Egypt?**	120	53.81	103	46.19	223
**Do you think it is a good idea for men to be concerned about the debate on FGM/C?**	112	50.22	111	49.78	223
**Do you think healthcare workers have a role to play in eliminating FGM/C?**	144	64.57	79	35.43	223
**a) It makes the practice safer**	114	51.12	109	48.88	223
**b) It is a way of encouraging FGM/C**	144	64.57	79	35.43	223
**c) It should be stopped**	82	36.77	141	63.23	223
**Practice**	n	%	n	%	n
**Was FGM/C practiced in your family/household?**	102	45.74	121	54.26	223
**Is FGM/C still being practiced in your family/household?**	108	48.43	115	51.57	223
**If you have a daughter in the future, do you intend to circumcise her?**	138	61.88	85	38.12	223
**As a health care provider, have you ever performed FGM/C?**	9	4.04	214	95.96	223
**Reasons to support**	n	%	n	%	n
**Do you support performing the FGM/C procedure**	134	60.09	89	39.91	223
**It is a mandatory religious practice**	134	60.09	89	39.91	223
**It is a deeply rooted cultural practice**	130	58.30	93	41.70	223
**It reduces sexual feelings**	169	75.78	54	24.22	223
**It is a rite of passage for girls into womanhood**	129	57.85	94	42.15	223
**It is a good practice**	133	59.64	90	40.36	223
**It helps to maintain their virginity for their husband**	129	57.85	94	42.15	223
**It reduces the rate of prostitution**	131	58.74	92	41.26	223

**Table 3:** Comparison based on the place of residence of the respondents and the p-value for the association based on the place of residence.

			Residence					
			Urban (n = 111)		Rural (n = 112)			
			Count	Column N %	Count	Column N %	X^2^	p value
**Reasons given by people to support FGM**	Do you support performing the FGM procedure	Yes	32	28.80%	102	91.10%	90.059	<0.001
		No	79	71.20%	10	8.90%		
	It is a mandatory religious practice	Yes	32	28.80%	102	91.10%	90.059	<0.001
		No	79	71.20%	10	8.90%		
	It is a deeply rooted cultural practice	Yes	29	26.10%	101	90.20%	94.079	<0.001
		No	82	73.90%	11	9.80%		
	It reduces sexual feelings	Yes	32	28.80%	102	91.10%	90.059	<0.001
		No	79	71.20%	10	8.90%		
	It is a rite of passage for girls into womanhood	Yes	29	26.10%	100	89.30%	91.203	<0.001
		No	82	73.90%	12	10.70%		
	It is a good practice	Yes	32	28.80%	101	90.20%	87.172	<0.001
		No	79	71.20%	11	9.80%		
	It helps to maintain their virginity for their husband	Yes	29	26.10%	100	89.30%	91.203	<0.001
		No	82	73.90%	12	10.70%		
	It reduces the rate of prostitution	Yes	32	28.80%	99	88.40%	81.612	<0.001
		No	79	71.20%	13	11.60%		
**Potenital consquences of FGM**	Transmission of infectious diseases	Yes	70	63.10%	20	17.90%	47.331	<0.001
		No	41	36.90%	92	82.10%		
	Bleeding	Yes	70	63.10%	20	17.90%	47.331	<0.001
		No	41	36.90%	92	82.10%		
	Health problems	Yes	70	63.10%	20	17.90%	47.331	<0.001
		No	41	36.90%	92	82.10%		
	Difficulty during delivery	Yes	71	64.00%	21	18.80%	47.025	<0.001
		No	40	36.00%	91	81.30%		
	Reduction of sexual feelings	Yes	67	60.40%	20	17.90%	42.328	<0.001
		No	44	39.60%	92	82.10%		
	Affects the health and welfare of women and girls	Yes	68	61.30%	20	17.90%	43.963	<0.001
		No	43	38.70%	92	82.10%		
	Difficult penetration during sex	Yes	67	60.40%	20	17.90%	42.328	<0.001
		No	44	39.60%	92	82.10%		
	No consequences	Yes	68	61.30%	21	18.80%	42.011	<0.001
		No	43	38.70%	91	81.30%		
	Has seen a girl with complications after FGM	Yes	71	64.00%	20	17.90%	49.064	<0.001
		No	40	36.00%	92	82.10%		
**Attitude towards FGM**	Do you think that the practice of FGM should continue?	Yes	46	41.40%	95	84.80%	45.122	<0.001
		No	65	58.60%	17	15.20%		
	Do you think that girls that have not undergone FGM should be discriminated?	Yes	63	56.80%	17	15.20%	41.894	<0.001
		No	48	43.20%	95	84.80%		
	Do you think that the practice of FGM can ever be eliminated in Egypt?	Yes	57	51.40%	63	56.30%	0.538	0.46
		No	54	48.60%	49	43.80%		
	Do you think it is a good idea for men to be concerned on the debate on FGM?	Yes	61	55.00%	51	45.50%	1.979	0.16
		No	50	45.00%	61	54.50%		
	Do you think HCP workers have a role to play in eliminating FGM?	Yes	60	54.10%	54	48.20%	0.761	0.38
		No	51	45.90%	58	51.80%		
	a) It makes the practice safer	Yes	71	64.00%	73	65.20%	0.036	0.85
		No	40	36.00%	39	34.80%		
	b) It is a way of encouraging FGM	Yes	71	64.00%	73	65.20%	0.036	0.85
		No	40	36.00%	39	34.80%		
	c) It should be stopped at all levels	Yes	74	66.70%	21	18.80%	52.346	<0.001
		No	37	33.30%	91	81.30%		
**Practice towards FGM**	Was FGM practiced in your family/household?	Yes	45	40.50%	57	50.90%	2.407	0.121
		No	66	59.50%	55	49.10%		
	Is FGM practiced in your family/household?	Yes	53	47.70%	55	49.10%	0.041	0.84
		No	58	52.30%	57	50.90%		
	If you have a daughter in the future, do you intend to circumcise her?	Yes	49	44.10%	89	79.50%	29.484	<0.001
		No	62	55.90%	23	20.50%		
	As a health care provider, have you ever carried out FGM/C on a girl?	Yes	4	3.60%	5	4.50%	0.107	0.74
		No	107	96.40%	107	95.50%		

**Table 4:** Comparison based on the religion of the respondents and the p-value for the association based on the religion reported.

			Religion					
			Muslim (n = 190)		Christian (n = 33)			
			Count	Column N %	Count	Column N %	X^2^	p value
**Reasons given by people to support FGM**	Do you support performing the FGM procedure	Yes	110	57.90%	24	72.70%	2.579	0.11
		No	80	42.10%	9	27.30%		
	It is a mandatory religious practice	Yes	110	57.90%	24	72.70%	2.579	0.11
		No	80	42.10%	9	27.30%		
	It is a deeply rooted cultural practice	Yes	106	55.80%	24	72.70%	3.318	0.07
		No	84	44.20%	9	27.30%		
	It reduces sexual feelings	Yes	110	57.90%	24	72.70%	2.579	0.11
		No	80	42.10%	9	27.30%		
	It is a rite of passage for girls into womanhood	Yes	105	55.30%	24	72.70%	3.517	0.06
		No	85	44.70%	9	27.30%		
	It is a good practice	Yes	109	57.40%	24	72.70%	2.755	0.09
		No	81	42.60%	9	27.30%		
	It helps to maintain their virginity for their husband	Yes	105	55.30%	24	72.70%	3.517	0.66
		No	85	44.70%	9	27.30%		
	It reduces the rate of prostitution	Yes	107	56.30%	24	72.70%	3.125	0.08
		No	83	43.70%	9	27.30%		
**Potenital consquences of FGM**	Transmission of infectious diseases	Yes	75	39.50%	15	45.50%	0.418	0.52
		No	115	60.50%	18	54.50%		
	Bleeding	Yes	75	39.50%	15	45.50%	0.418	0.52
		No	115	60.50%	18	54.50%		
	Health problems	Yes	75	39.50%	15	45.50%	0.418	0.52
		No	115	60.50%	18	54.50%		
	Difficulty during delivery	Yes	76	40.00%	16	48.50%	0.835	0.36
		No	114	60.00%	17	51.50%		
	Reduction of sexual feelings	Yes	71	37.40%	16	48.50%	1.460	0.23
		No	119	62.60%	17	51.50%		
	Affects the health and welfare of women and girls	Yes	74	38.90%	14	42.40%	0.142	0.71
		No	116	61.10%	19	57.60%		
	Difficult penetration during sex	Yes	73	38.40%	14	42.40%	0.189	0.66
		No	117	61.60%	19	57.60%		
	No consequences	Yes	73	38.40%	16	48.50%	1.187	0.28
		No	117	61.60%	17	51.50%		
	Has seen a girl with complications after FGM	Yes	76	40.00%	15	45.50%	0.346	0.57
		No	114	60.00%	18	54.50%		
**Attitude towards FGM**	Do you think that the practice of FGM should continue?	Yes	118	62.10%	23	69.70%	0.697	0.4
		No	72	37.90%	10	30.30%		
	Do you think that girls that have not undergone FGM should be discriminated?	Yes	70	36.80%	10	30.30%	0.523	0.47
		No	120	63.20%	23	69.70%		
	Do you think that the practice of FGM can ever be eliminated in Egypt?	Yes	108	56.80%	12	36.40%	4.744	0.03
		No	82	43.20%	21	63.60%		
	Do you think it is a good idea for men to be concerned on the debate on FGM?	Yes	95	50.00%	17	51.50%	0.026	0.87
		No	95	50.00%	16	48.50%		
	Do you think HCP workers have a role to play in eliminating FGM?	Yes	97	51.10%	17	51.50%	0.002	0.96
		No	93	48.90%	16	48.50%		
	a) It makes the practice safer	Yes	122	64.20%	22	66.70%	0.074	0.79
		No	68	35.80%	11	33.30%		
	b) It is a way of encouraging FGM	Yes	122	64.20%	22	66.70%	0.074	0.79
		No	68	35.80%	11	33.30%		
	c) It should be stopped at all levels	Yes	84	44.20%	11	33.30%	1.360	0.24
		No	106	55.80%	22	66.70%		
**Practice towards FGM**	Was FGM practiced in your family/household?	Yes	84	44.20%	18	54.50%	1.210	0.27
		No	106	55.80%	15	45.50%		
	Is FGM practiced in your family/household?	Yes	90	47.40%	18	54.50%	0.580	0.45
		No	100	52.60%	15	45.50%		
	If you have a daughter in the future, do you intend to circumcise her?	Yes	117	61.60%	21	63.60%	0.050	0.82
		No	73	38.40%	12	36.40%		
	As a health care provider, have you ever carried out FGM/C on a girl?	Yes	8	4.20%	1	3.00%	0.101	0.75
		No	182	95.80%	32	97.00%		

**Table 5:** Comparison based on the gender of the respondents and the p-value for the association based on gender.

			Gender					
			Male (n = 81)		Female (n = 142)			
			Count	Column N %	Count	Column N %	X^2^	p value
**Reasons given by people to support FGM**	Do you support performing the FGM procedure	Yes	47	58.00%	87	61.30%	0.226	0.63
		No	34	42.00%	55	38.70%		
	It is a mandatory religious practice	Yes	47	58.00%	87	61.30%	0.226	0.63
		No	34	42.00%	55	38.70%		
	It is a deeply rooted cultural practice	Yes	43	53.10%	87	61.30%	1.42	0.23
		No	38	46.90%	55	38.70%		
	It reduces sexual feelings	Yes	47	58.00%	87	61.30%	0.2226	0.63
		No	34	42.00%	55	38.70%		
	It is a rite of passage for girls into womanhood	Yes	44	54.30%	85	59.90%	0.649	0.42
		No	37	45.70%	57	40.10%		
	It is a good practice	Yes	47	58.00%	86	60.60%	0.138	0.71
		No	34	42.00%	56	39.40%		
	It helps to maintain their virginity for their husband	Yes	43	53.10%	86	60.60%	1.183	0.28
		No	38	46.90%	56	39.40%		
	It reduces the rate of prostitution	Yes	47	58.00%	84	59.20%	0.027	0.87
		No	34	42.00%	58	40.80%		
**Potenital consquences of FGM**	Transmission of infectious diseases	Yes	44	54.30%	46	32.40%	10.302	0.001
		No	37	45.70%	96	67.60%		
	Bleeding	Yes	44	54.30%	46	32.40%	10.302	0.001
		No	37	45.70%	96	67.60%		
	Health problems	Yes	44	54.30%	46	32.40%	10.302	0.001
		No	37	45.70%	96	67.60%		
	Difficulty during delivery	Yes	45	55.60%	47	33.10%	10.733	0.001
		No	36	44.40%	95	66.90%		
	Reduction of sexual feelings	Yes	41	50.60%	46	32.40%	7.199	0.007
		No	40	49.40%	96	67.60%		
	Affects the health and welfare of women and girls	Yes	42	51.90%	46	32.40%	8.174	0.004
		No	39	48.10%	96	67.60%		
	Difficult penetration during sex	Yes	42	51.90%	45	31.70%	8.812	0.003
		No	39	48.10%	97	68.30%		
	No consequences	Yes	41	50.60%	48	33.80%	6.081	0.014
		No	40	49.40%	94	66.20%		
	Has seen a girl with complications after FGM	Yes	44	54.30%	47	33.10%	9.617	0.002
		No	37	45.70%	95	66.90%		
**Attitude towards FGM**	Do you think that the practice of FGM should continue?	Yes	50	61.70%	91	64.10%	0.123	0.73
		No	31	38.30%	51	35.90%		
	Do you think that girls that have not undergone FGM should be discriminated?	Yes	29	35.80%	51	35.90%	0	0.97
		No	52	64.20%	91	64.10%		
	Do you think that the practice of FGM can ever be eliminated in Egypt?	Yes	38	46.90%	82	57.70%	2.435	0.12
		No	43	53.10%	60	42.30%		
	Do you think it is a good idea for men to be concerned on the debate on FGM?	Yes	45	55.60%	67	47.20%	1.446	0.23
		No	36	44.40%	75	52.80%		
	Do you think HCP workers have a role to play in eliminating FGM?	Yes	43	53.10%	71	50.00%	0.197	0.66
		No	38	46.90%	71	50.00%		
	a) It makes the practice safer	Yes	56	69.10%	88	62.00%	1.157	0.28
		No	25	30.90%	54	38.00%		
	b) It is a way of encouraging FGM	Yes	56	69.10%	88	62.00%	1.157	0.28
		No	25	30.90%	54	38.00%		
	c) It should be stopped at all levels	Yes	48	59.30%	47	33.10%	14.436	<0.001
		No	33	40.70%	95	66.90%		
**Practice towards FGM**	Was FGM practiced in your family/household?	Yes	42	51.90%	60	42.30%	1.915	0.17
		No	39	48.10%	82	57.70%		
	Is FGM practiced in your family/household?	Yes	35	43.20%	73	51.40%	1.388	0.24
		No	46	56.80%	69	48.60%		
	If you have a daughter in the future, do you intend to circumcise her?	Yes	38	46.90%	100	70.40%	12.085	0.001
		No	43	53.10%	42	29.60%		
	As a health care provider, have you ever carried out FGM/C on a girl?	Yes	6	7.40%	3	2.10%	3.733	0.76
		No	75	92.60%	139	97.90%		

Table 3 shows a comparison between the place of residence of the respondents. The table also includes the p-value for the result of the association test used as described in the methodology. Among rural and urban groups, significant differences were found. Notably, all questions about consequences and the reasons to support them showed a statistically significant association. Furthermore, a significantly higher agreement was found among HCPs in rural areas compared to those in urban areas that if they have a daughter in the future, they will intend to circumcise her.

As seen in Table 1, 60% of the respondents believed that FGM/C is a mandatory religious practice. Regarding the breakdown, around 72% of the Christian respondents agreed, while only 58% of the Muslim respondents agreed. Most questions did not result in a statistically significant association, except for a lower agreement that FGM has no consequences and a high agreement that FGM reduces sexual feelings in Christian respondents. Also, a higher agreement among Muslim respondents that FGM/C can be eliminated in Egypt. The full breakdown by religious belief can be seen in Table 4.

Table 5 shows the comparison between the gender of respondent and their knowledge, attitudes, and practices about FGM/C. The attitudes domain showed no significant relation with the gender of respondents, while most of the knowledge domain showed a significant association. A significantly higher agreement was found among women compared to men that if they have a daughter in the future, they will intend to circumcise her. The full details can be found in Table 5.

## Discussion

This study’s results demonstrate that despite the various efforts to ban FGM/C in countries worldwide, attitudes supporting FGM/C are still far from being eradicated and have hardly changed over the past years. What was interesting during this study’s literature review was that from 1978 to 1995, there was only one study investigating attitudes toward FGM/C. Since the 2000s, studies exploring attitudes towards FGM/C have increased after it was recognized as an essential problem and gained increased attention worldwide [36, 37].

UNICEF’s 2016 report highlights that HCPs perform FGM/C due to erroneous information [1]. Therefore, this study aimed to understand the KAP of HCP related to FGM/CA study was conducted in the Valencian region of Spain by González-Timoneda et al., who assessed knowledge, attitude, and practice on FGM/C among primary healthcare professionals. Of the 321 responses received, more than 70% of the respondents were women, ranging between 22 and 68 years old. Regarding reasons, they observed that 44.2% of the general practitioner attributed FGM/C to religion and 33.3% to traditions and customs [38]. Our findings were higher, where around 60% attributed it to religion and 58% to traditions and customs.

In the same context, Marcusán AK et al. conducted a cross-sectional descriptive study designed to understand the knowledge, attitudes, and practices regarding FGM/C among HCPs in Gambia. They analyzed a stratified sample of 1,288 HCPs, including HCPs and students, throughout the Gambia. Most HCPs surveyed, 96.5%, cited deeply rooted cultural practice as the main reason for FGM/C persistence in the country. In comparison, 78.5% revealed it reduces sexual feelings, and these results are comparable to ours, where around 75% agreed on the same [39].

Our findings suggest that many HCPs do not consider the adverse consequences of FGM/C and insist on continuing this practice for sociocultural reasons rather than health-related reasons of the young women and girls, as 63% believed the practice should continue. Around 60% believe it is a religious and cultural practice. This agrees with the increase in FGM practices from the 2015 EDHS survey, where 42.4% reported being circumcised by an HCP vs. 47% in the 2022 EFHS survey [26]. Unfortunately, this reflects concerning trends of continuing the practice of FGM medicalization, stemming from deeply rooted cultural and social concerns among HCPs. In the present study, 28.8% of the respondents in urban areas and 91.1% in rural areas supported the procedure.

These results agreed with the findings of the UNICEF, which reported that FGM/C is found more often among those living in rural areas and that the most significant disparities between urban and rural residents were reported in Egypt, as was described in the 2014 EDHS, urban residents have higher educational levels as they are more likely to have attended school and/or remained in school for a more extended period than rural residents. Also, there are considerable differences reported in the wealth index distributions by residence, where the larger proportion of those living in urban settings in Egypt belong to the two highest wealth quintiles, and more of the rural population are in the two lowest wealth index groups, as shown in the 2014 EDHS. Eritrea, Ethiopia, and Guinea [40]. Egypt and Guinea, countries with a high prevalence of FGM/C, have nearly similar values: 57% (urban) and 82% (rural), and 55% (urban) and 75% (rural) were reported, respectively. However, the opposite is present in Nigeria, where 16% of the urban population supports FGM/C versus 9% of rural residents (199). In Iraq, FGM/C is most common among the richest wealth quintile, and those with more education in Sudan have a higher prevalence [41]. This is aligned with our finding, where around 80% of the rural respondents said that they intend to mutilate their daughters, in contrast to 44% of the urban respondents.

Also, our results revealed that 91% from rural backgrounds support FGM, in contrast to 27% from urban backgrounds, and 84% from rural backgrounds agree that FGM should continue due to cultural and religious reasons. This agrees with Balde et al., who explored the attitudes of HCP related to FGM/C, its medicalization, and how the health sector can play a role in addressing this practice. They reported that some HCPs in rural areas stated that they would agree to excision/perform FGM because it is a custom or because of a lack of information. Through the interviews, they found that some midwives in rural Faranah believe that the excision is essential as it would protect the girls from being stigmatized, reflecting ambivalence about FGM [42]. Moreover, in agreement with our findings, Abolfotouh et al., assessed the awareness and predictors of FGM/C in young Egyptian health advocates. They noticed that urban areas had higher average knowledge of adverse health consequences and attitude towards discontinuation scores (54.18 and 77.85, respectively), which indicate a more positive attitude toward discontinuation of FGM/C [43].

Concerning participants’ religion and reasons for supporting FGM/C, in the current study, approximately 73% of Christian HCP and 60% of Muslims supported the procedures. Despite being higher, it did not show a significant association as per the p-value of the chi-squared test. Our results agreed with Oladeji et al., assessing healthcare workers’ knowledge, attitude, and practice on FGM/C practices in the Somali region of Ethiopia. They reported that most health workers mentioned cultural practice as the primary reason for FGM/C practice in the region much more than being a religious practice [44]. Similarly, Marcusan et al.’s cross-sectional study in Gambia assessed the knowledge, attitudes, and practices among HCPs working in rural settings. They included 468 HCPs, including all nurse cadres and midwives. About 60% of the health workers reported religion as a reason for FGM/C practice [45]. Other factors, such as race, culture, and beliefs, may substantially impact these responses and the incorrect association of this behavior with religion since several individuals misinterpret their religions. This is also aligned with the different religious authorities’ views where both the Islamic and Christian authorities have condemned or confirmed that no religious text do support such practice [31, 32].

Regarding the gender of participants and the reasons given to support FGM/C, in the current study, women female and men participants were equally supportive of the performance of the procedure (61.3%, 58.0%), respectively. The most commonly cited reasons for the performance of the procedure, according to women female participants, were: reduction of sexual feelings (73.9%), mandatory religious practice (61.3%), and deeply rooted cultural practice (61.3%), as compared to male menicipants who noted: reduction of sexual feelings (79%), mandatory religious practice (58%), good practice (58%) and reduction of the rate of prostitution (58%), as the most common reasons.

Our results agree with Mostafa et al., who explored the knowledge, beliefs, and attitudes of three hundred and thirty 5th year medical students at Alexandria University towards FGM/C. Their study reported that 50% of the surveyed medical students were against this practice. Both men and women were similar with no significant differences and mentioned they could contribute to stopping this practice in their future careers. Nevertheless, 31.9% expressed their intention to subject their future daughters to circumcision [46].

The findings of our study challenge, Abdelmoaty et al.’s observation that the surveyed sample expressed a negative attitude towards FGM/C, refusing this practice to continue; women showed more opposition to FGM/C than men [47]. The deviation from our findings may be attributed to the fact that their participants were from one center, as they included Egyptian Medical students at Kasr Al Ainy Medical School, as medical students in urban areas are more likely to be aware of the negative impact of FGM/C.

Although this study was conducted among HCPs, knowledge of potential complications was low among rural participants compared to urban areas (18.8%, and 64%, respectively). A significant number of men participants believed that FGM/C increases the transmission of infectious diseases, causes bleeding and different health problems, and causes painful penetration during sex and difficulty during delivery compared to female participants. However, more Christian participants were aware of FGM/C consequences than Muslim respondents. The above findings reflect the need for education and guidelines relevant to FGM/C in basic medical training and continuing medical education.

Abolfotouh et al., supported our findings. They reported that 37.5% of their responders revealed that FGM/C could lead to complications during childbirth [40]. Similarly, in a study by Ali AAA, 64% of the respondents believed that FGM/C decreases sexual pleasure, and 33% reported that it increases the transmission of infectious diseases [48].

On another note, this study revealed that a significant number of rural HCP, compared to those from urban areas, believed that FGM/C should continue to be practiced and girls who have not undergone FGM/C should be discriminated against. Moreover, a significantly higher agreement was found among HCPs in rural areas compared to those in urban areas that if they have a daughter in the future, they intend to circumcise her. This latter finding is highly reflective of participants’ attitudes towards the practice.

Our results are supported by Balde et al., who reported that some midwives in rural Faranah believed it is necessary to circumcise girls to keep them from being stigmatized, according to the results of interviews [39]. Also, in agreement with our findings, Marcusan et al., reported that a significant proportion of Gambian HCPs included in the study, working in rural areas supported the continuation of FGM/C (42.5%) and plan to have it done to their daughters (47.2%) [42].

In the present study, the gender and religion of the participants had no significant effect on the respondent’s attitude to the practice of FGM/C. However, many female respondents desired to circumcise their future daughters compared to men.

Concerning respondents’ attitudes toward the medicalization of the practice, approximately 65% of participants believed that HCP could play a role in eliminating the practice, and around 51% reported that this makes the practice safer. Interestingly, a similar percentage of participants believe that the medicalization of the procedure encourages this practice. Nevertheless, a significantly higher agreement was found among HCPs in urban areas compared to those in rural areas that FGM/C should be stopped. Our results agreed with Mostafa et al., who reported that gaps were identified in knowledge about the prevalence of FGM/C, FGM/C types, complications, and the ethical and legal aspects. In their study, two hundred forty (73.2%) medical students favored its "medicalization" to reduce pain and risks to health [43]. Contrary to our findings, a study by Relph et al., observed that only 8.9% of HCPs agreed that the procedure should be medicalized to reduce the associated morbidity [49].

Finally, this study has been conducted among HCPs from various specialties. This study found a considerable lack of knowledge among the participants about the consequences of the procedure and, hence the willingness to circumcise their future daughters, which is included in the medical school curriculum. Many medical graduates may serve as general practitioners in Egypt immediately after graduation for up to 2 years in rural areas. They may be exposed to requests for FGM, so they need to be better educated. This could be attributed to the fact that most of the consequences of FGM appear later on in life, years after the operation; thus, women will not be able to relate the complications to FGM.

Significant differences were found among rural and urban groups regarding the support of the FGM/C procedure and whether they considered FGM/C to be mandatory by religion. A significantly higher agreement was found among HCPs in rural areas compared to those in urban areas regarding its cultural roots, the rite of passage to womanhood, maintaining virginity, and reducing the rate of prostitution. Comparing the gender of respondents’ practice of FGM/C, a significantly higher agreement was found among women compared to men that if they have a daughter in the future, they intend to circumcise her.

A considerable number of the respondents believed that medicalization could help reduce the risk of complications. This is partially true as it might reduce immediate/short-term complications but does not prevent long-term complications. HCPs can take a leading role in raising the awareness of women and combating FGM. Hence, educational materials and policies must be developed to raise awareness and prevent this harmful practice.

Ending FGM/C is a significant public health challenge due to its deep cultural roots [50]. Mass education is urgently needed for healthcare providers and the general public to mobilize the community against this practice. Changing community perceptions and dispelling myths associated with FGM/C is crucial for ending it. Families struggle to abandon the practice without broader community support. Advocates stress the importance of public education, women’s empowerment, urbanization, and informing the public about the risks of FGM/C in changing attitudes. Legislation alone is not sufficient to eliminate FGM/C, as shown by evidence. Religious institutions and leaders also have a vital role in convincing their followers that FGM/C has harmful health consequences.

An important initiative to change social norms and perceptions related to FGM involves the creation of the film "Between Two Seas." This movie is a collaborative effort between the National Council for Women and UN Women Egypt, recognizing the power of cinema to influence social norms and foster dialogue for the betterment of women’s rights. The film, written by Mariam Naoum, directed by Anas Tolba, and produced by Axeer, received support from the Government of Japan and the United States Agency for International Development (USAID). "Between Two Seas" was released in theaters in 2019 and is now available on Netflix. It has received 22 international awards after participating in various international and regional film festivals. The National Council for Women and UN Women Egypt took the additional step of organizing free public screenings in different governorates, aiming to change people’s perceptions on various issues, including FGM and domestic violence. These screenings also included interactive discussions led by NCW Rapporteurs to engage women and men in meaningful conversations about these important topics [51].

This study has limitations, including being conducted in person using a self-administered questionnaire, which might have affected the participants’ responses. Additionally, only a very small sample performed FGM before, so it would be interesting to understand such groups’ knowledge, attitudes, and practices. For future research, a more in-depth methodology, like in-depth interviews or Focus Group Discussions, could be used to explore the problem’s core further. Also, the study was implemented in two public hospitals; therefore, the authors recommend further studies on a larger sample size and a large geographical scale to emphasize our conclusion. All participants were from large governmental hospitals, excluding private facilities, which are an essential part of the health system in Egypt [52, 53]. Thus, these findings may have limited generalizability all over the country.

## Conclusion and recommendations

The prevalence of FGM remains a significant concern in Egyptian society, as indicated by the study’s findings showing substantial support for the practice among the examined HCPs and a tendency for medicalization. These results emphasize the need to develop effective strategies for empowering HCPs to prevent and manage FGM while discouraging its medicalization. However, it is vital to design capacity-building programs that consider cultural and gender sensitivity since education alone may not be sufficient to address the issue. The complexity of the problem is evident in the conflicting stances taken by the HCPs in the study, necessitating acknowledgment and resolution of these differing perspectives. From a gender perspective, achieving social change requires the active involvement of both men and women, underscoring the urgency of investigating women’s knowledge, attitudes, and practices in greater depth.

HCPs possess the potential to become instrumental figures in the fight against FGM/C due to their integration and credibility within the community. Given their role as primary responders to FGM/C-related complications, their engagement becomes particularly critical in rural areas where the practice is more prevalent and access to quality healthcare is limited. Integrating them into the community makes them pivotal in preventing and effectively managing the consequences. Consequently, bridging the gap in medical school curricula through informal learning activities and ongoing medical education programs focused on sexual and reproductive health and human rights becomes paramount. Recognizing that legislative measures and law enforcement alone are insufficient to eradicate FGM/C from society, more significant efforts should be made to raise public awareness, particularly among HCPs.

By incorporating it early in medical curricula, we recommend providing medical students and HCPs with comprehensive scientific knowledge about FGM/C, with a particular emphasis on its detrimental effects on reproductive health. Organizing community-based health awareness campaigns becomes imperative to sensitize the Egyptian population about this harmful practice. It is crucial to acknowledge that behavioral change is a gradual process, making each activity, campaign, initiative, and policy change essential in eradicating FGM/C from society.

Furthermore, it is important to encourage the establishment of community dialogues that foster trust between older and younger generations, enabling them to address harmful practices like FGM jointly. Utilize intergenerational dialogues, educational initiatives, and social mobilization sessions as effective tools. Furthermore, it advocates for sustained, long-term interventions that actively involve community members, including religious and community leaders, in challenging and reshaping the social norms associated with FGM/C. This would be further strengthened by enforcing relevant laws, spreading awareness through rural female community leaders who engage with women directly, and utilizing films and TV series as recommended actions. Encourage cooperation between medical and religious organizations to raise awareness about the medicalization of FGM/C, targeting both medical professionals and religious leaders and involving the Ministry of Education in integrating information about the risks of the practice into educational curricula.

We believe a collaborative multi-stakeholder approach is the only way to address the problem.

## Supporting information

S1 Questionnaire FGM/C–Questionnaire. (DOCX)

## References

[1] United Nations Children’s Fund (UNICEF). Female Genital Mutilation/Cutting: A Global Concern. UNICEF. 2016. https://www.unicef.org/media/files/FGMC_2016_brochure_final_UNICEF_SPREAD.pdf

[2] World Health Organization (WHO). Eliminating female genital mutilation: An interagency statement—OHCHR, UNAIDS, UNDP, UNECA, UNESCO, UNFPA, UNHCR, UNICEF, UNIFEM, WHO. WHO. 2018. https://iris.who.int/bitstream/handle/10665/43839/9789241596442_eng.pdf?sequence=1

[3] Elduma A. Female Genital Mutilation in Sudan. Open Access Maced J Med Sci. 2018;6(2):430–434. doi: 10.3889/oamjms.2018.099 29531618 PMC5839462

[4] UNFPA-UNICEF Joint Programme on Female Genital Mutilation/Cutting (2017) 17 Ways to End FGM/C, p.49. Available at https://www.unfpa.org/sites/default/files/pub-pdf/17ways-web.pdf.

[5] Hassanin I, Saleh R, Bedaiwy A, Peterson R, Bedaiwy M. Prevalence of female genital cutting in Upper Egypt: 6 years after enforcement of prohibition law. Reprod Biomed Online. 2008;16 Suppl 1:27–31. doi: 10.1016/s1472-6483(10)60396-3 18348787

[6] El-Gibaly O, Aziz M, Abou Hussein S. Health care providers’ and mothers’ perceptions about the medicalization of female genital mutilation or cutting in Egypt: a cross-sectional qualitative study. BMC Int Health Hum Rights 19, 26. 2019. doi: 10.1186/s12914-019-0202-x 31455345 PMC6712689

[7] Marea C, Warren N, Glass N, Johnson-Agbakwu C, Perrin N. Factors Associated with Health Care Provider Attitudes, and Confidence for the Care of Women and Girls Affected by Female Genital Mutilation/Cutting. Health Equity. 2021 May 19;5(1):329–337. doi: 10.1089/heq.2020.0130 34036217 PMC8140356

[8] WHO. Global strategy to stop health-care providers from performing female genital mutilation. 2010.

[9] Serour G. Medicalization of female genital cutting/mutilation. Afr J Urol. 2013;19(3):145–149.

[10] Kimani S, Shell-Duncan B. Medicalized female genital mutilation/cutting: contentious practices and persistent debates. Curr Sex Health Rep. 2018;10(1):25–34. doi: 10.1007/s11930-018-0140-y 29541004 PMC5840226

[11] Shell-Duncan B, Njue C, Moore Z. The medicalization of female genital mutilation /cutting: what do the data reveal? Evidence to end FGM/C: research to help women thrive. New York: Population Council; 2017.

[12] UNICEF, Gupta G. Female Genital Mutilation/Cutting: a statistical overview and exploration of the dynamics of change. Reproductive Health Matters. 2013;21(42):184–190.

[13] European Union (EU). Briefing—FGM in a humanitarian context, EU, Brussels, Belgium 2018. https://www.endfgm.eu/content/documents/reports/End-FGM-EU-Briefing-FGM-in-a-Humanitarian-Context.pdf

[14] Baillot H, Murray N, Connelly E, Howard N. Addressing female genital mutilation in Europe: a scoping review of approaches to participation, prevention, protection, and provision of services. Int J Equity Health. 2018;17(1):21. doi: 10.1186/s12939-017-0713-9 29422053 PMC5806373

[15] Van Baelen L, Ortensi L, Leye E. Estimates of first-generation women and girls with female genital mutilation in the European Union, Norway and Switzerland. Eur J Contracept Reprod Health Care. 2016;21(6):474–482. doi: 10.1080/13625187.2016.1234597 27652839

[16] Brown K, Beecham D, Barrett H. The Applicability of Behaviour Change in Intervention Programmes Targeted at Ending Female Genital Mutilation in the EU: Integrating Social Cognitive and Community Level Approaches. Obstetrics and Gynecology International, vol. 2013, Article ID 324362, 12 pages, 2013. doi: 10.1155/2013/324362 23983698 PMC3745976

[17] EU. Ending Female Genital Mutilation: A Strategy for the European Union Institutions, EndFGM, Brussels, Belgium, 2010.

[18] European Parliament Combating Female Genital Mutilation in the EU: European Parliament Resolution of 24 March 2009 on Combating Female Genital Mutilation in the EU (2008/2071(INI)), European Parliament, Brussels, Belgium, 2009.

[19] WHO. Classification of female genital mutilation. WHO. 2015. [http://www.who.int/reproductivehealth/topics/FGM/C/overview/en/]. Last accessed on: 08/09/2015.

[20] Assaad M. Female Circumcision in Egypt: Social Implications, Current Research and Prospects for Change. Stud Family Plann. 1980;11(1):3–16. 7376234

[21] Chibber R, El-Saleh E, El Harmi J. Female circumcision: obstetrical and psychological sequelae continue unabated in the 21st century. J Matern Fetal Neonatal Med. 2011, 24 (6): 833–836. doi: 10.3109/14767058.2010.531318 21121711

[22] Vogt S, Mohmmed Zaid N, El Fadil Ahmed H, Fehr E, Efferson C. Changing cultural attitudes towards female genital cutting. Nature. 2016;538(7626):506–509. doi: 10.1038/nature20100 27732586

[23] Williams-Breault B. Eradicating Female Genital Mutilation/Cutting: Human Rights-Based Approaches of Legislation, Education, and Community Empowerment. Health Hum Rights. 2018;20(2):223–233. 30568416 PMC6293358

[24] The Ministry of Health and Population Egypt, El-Zanaty and Associates Egypt, ICF International. 2015. The Egypt Demographic and Health Survey 2014. Cairo, Egypt: Ministry of Health and Population and ICF International. https://dhsprogram.com/publications/publication-FR302-DHS-Final-Reports.cfm

[25] UNICEF. Female Genital Mutilation in Egypt: Recent trends and projections, UNICEF, New York, 2020

[26] Central Agency for Public Mobilization and Statistics (CAPMAS). Egyptian Family Health Survey, 2022.

[27] UNICEF. Egypt Data Snapshot—Issue 2, June 2019.

[28] Shell-Duncan B. The medicalization of female "circumcision": harm reduction or promotion of a dangerous practice? Soc Sci Med. 2001;52(7):1013–1028. doi: 10.1016/s0277-9536(00)00208-2 11266046

[29] Ministry of State for Population and United Nations Egypt (2016) The National FGM Abandonment Strategy 2016–2020. http://www.gbvprojectegypt.com/assets/documents/resources/fgm-national-strategy.pdf.

[30] Human Rights Watch. New Penalties for Female Genital Mutilation, Egypt. Human Rights Watch. 2016. https://www.hrw.org/news/2016/09/09/egypt-new-penalties-female-genital-mutilation

[31] FGM- CRI. Too Many. Egypt: The law and FGM/C. 2018;12(1):4–6. https://www.28toomany.org/media/uploads/Law%20Reports/egypt_law_report_v1_(june_2018).pdf

[32] El-Damanhoury I. The Jewish and Christian view on female genital mutilation. African Journal of Urology 19.3 (2013): 127–129.

[33] Saad Menna (2018) ‘Female Genital Mutilation is declared religiously forbidden in Islam’, Egypt Today, 31 May. Available at https://www.egypttoday.com/Article/2/51304/Female-Genital-Mutilation-is-declared-religiously-forbidden-in-Islam.

[34] Epi-calc 2000 software. www.brixtonhealth.com/epicalc.html

[35] Goodyear M, Krleza-Jeric K, Lemmens T. The Declaration of Helsinki. BMJ. 2007 Sep 29;335(7621):624–5. doi: 10.1136/bmj.39339.610000.BE 17901471 PMC1995496

[36] Gele A, Bø B, Sundby J. Have we made progress in Somalia after 30 years of interventions? Attitudes toward female circumcision among people in the Hargeisa district. BMC Res. Notes 2013, 6, 122. doi: 10.1186/1756-0500-6-122 23537232 PMC3614471

[37] Jahangiry L, Pashaei T, Ponnet K. Attitudes toward Female Genital Mutilation/Circumcision: A Systematic Review and Meta-Analysis. Healthcare (Basel). 2021 Sep 8;9(9):1184. doi: 10.3390/healthcare9091184 ; PMCID: PMC8466725. 34574958 PMC8466725

[38] González-Timoneda A, Ruiz Ros V, González-Timoneda M, Cano Sánchez A. Knowledge, attitudes and practices of primary healthcare professionals to female genital mutilation in Valencia, Spain: are we ready for this challenge? BMC health services research. 2018;18(1):579–81. doi: 10.1186/s12913-018-3396-z 30041654 PMC6057065

[39] Marcusán AK, Singla L, Secka D, Utzet M, Le Charles M. Female genital mutilation/cutting: changes and trends in knowledge, attitudes, and practices among health care professionals in the Gambia. Int J Women’s Health. 2016;8:103–5. 27110140 10.2147/IJWH.S102201 PMC4835126

[40] UNICEF. Female genital mutilation/cutting: a statistical exploration. UNICEF. 2005.

[41] Govender K, Cowden R, Nyamaruze P, Armstrong R, Hatane L. Beyond the disease: Contextualized implications of the COVID-19 pandemic for children and young people living in Eastern and Southern Africa. Front Public Health. 2020;8:504–506. doi: 10.3389/fpubh.2020.00504 33194933 PMC7604346

[42] Balde M O’Neill S, Sall A, Balde M, Soumah A, Diallo B, et al. Attitudes of health care providers regarding female genital mutilation and its medicalization in Guinea. PLoS One. 2021;16(5):998–1002. doi: 10.1371/journal.pone.0249998 33983949 PMC8118326

[43] Abolfotouh S, Ebrahim A, Abolfotouh M. Awareness and predictors of female genital mutilation/cutting among young health advocates. Int J Womens Health. 2015;7:259–69. doi: 10.2147/IJWH.S78664 25759602 PMC4346006

[44] Oladeji O, Farah A, Aden B. Knowledge, attitudes and practices of female genital mutilation among health care workers in Somali region of Ethiopia. Int J Community Med Public Health. 2021;8(9):4191–5.

[45] Kaplan A, Hechavarría S, Bernal M, Bonhoure I. Knowledge, attitudes and practices of female genital mutilation/cutting among health care professionals in The Gambia: a multiethnic study. BMC Public Health. 2013;13(1):851–5. doi: 10.1186/1471-2458-13-851 24040762 PMC3848660

[46] Mostafa S, El Zeiny N, Tayel S, Moubarak E. What do medical students in Alexandria know about female genital mutilation? East Mediterr Health J. 2006;12 Suppl 2:S78–92. 17361680

[47] Abdelmoaty A, Sabry H, Elamir R. Knowledge, Attitude and Intention to Future Practice of Female Genital Mutilation among Medical Students, Egypt. EFMJ. 2020;4(2):7–21.

[48] Ali A. Knowledge and attitudes of female genital mutilation among midwives in Eastern Sudan. Reproductive Health. 2012;9(1):23–5. doi: 10.1186/1742-4755-9-23 23020897 PMC3499224

[49] Relph S, Inamdar R, Singh H, Yoong W. Female genital mutilation/cutting: knowledge, attitude and training of health professionals in inner city London. Eur J Obstet Gynecol Reprod Biol. 2013;168(2):195–198. doi: 10.1016/j.ejogrb.2013.01.004 23434403

[50] Amin T, Moety A, Sabry H. Female Genital Mutilation: Egypt in focus. 2017. European Journal of Forensic Sciences. doi: 10.5455/ejfs.23650

[51] Women UN. National efforts continue to eliminate Female Genital Mutilation and reinforce a zero-tolerance culture. UN Women 2022. National efforts continue to eliminate Female Genital Mutilation and reinforce a zero-tolerance culture | UN Women–Egypt.

[52] Al Bahnasy R, Mohamed O, El-Shazly H, Abdel-Azeem A, Khedr R. The successes and the challenges of Egyptian Health Sector Reform Program. Menoufia Med J 2016;29:979–83.

[53] Gericke C, Britain K, Elmahdawy M, Elsisi G. Health System in Egypt. 2018. Health Care Systems and Policies. Health Services Research. Springer, New York, NY. 10.1007/978-1-4614-6419-8_7-1

