# Expression of Concern: Knowledge, attitudes, and practices of Female Genital Mutilation / Cutting among healthcare providers in two public hospitals in Egypt: A cross-sectional study

**DOI:** 10.1371/journal.pgph.0007200

**Published:** 2026-08-26

**Authors:** The PLOS Global Public Health Editors

Following publication of this article [1], concerns were raised about study design and data integrity. The *PLOS Global Public Health* Editors discussed the issues with the corresponding author and consulted a member of the journal’s Editorial Board. The Editors consider that several issues could not be fully resolved and issue this Expression of Concern to inform readers about study design limitations that impact the strength of support for the reported conclusions. Specific concerns and the input received about these issues are summarized below.

## The questionnaire

The questionnaire used in the study [1] is reported as adapted from a prior study of knowledge, attitudes and practices of female genital mutilation/cutting (FGM/C) conducted in Gambia [2], translated into Arabic, then back-translated and piloted among ten healthcare providers (HCPs) in a different public hospital to fit the Egyptian context. The consulted Editorial Board Member advised that

- The validity of the questionnaire in the Egyptian context is not adequately supported owing to the small sample size of ten HCPs and the absence of reported psychometric properties.
- There are several measurement‑validity concerns:
  ◦ The block on “reasons given by people who support FGM/C” follows immediately after the question asking whether the respondent supports the practice and uses binary yes/no responses to highly loaded statements (e.g., “good practice,” “reduces prostitution,” “maintains virginity”). This structure risks conflating whether respondents are reporting others’ beliefs or endorsing them personally and may prime for particular responses.
  ◦ The instrument offers limited neutral or non-endorsed pathways (e.g., lacks explicit “don’t know,” “other,” or “none” options), reducing construct validity for nuanced attitudes.
- The “medicalization” item includes the selectable statement “it makes the practice safer”, which may normalize a known misconception that WHO warns may unintentionally legitimize FGM and undermine abandonment efforts [3]; therefore, this item requires careful contextualization.

The corresponding author noted that the questionnaire is adapted from a previously validated instrument [2] and that the piloting among ten healthcare providers was intended to assess linguistic clarity, cultural appropriateness, face validity, and feasibility of administration, rather than to conduct psychometric revalidation. The corresponding author noted that this pilot sample size does not support formal psychometric evaluation and that the absence of reported psychometric properties should be acknowledged as a limitation of the study. The Arabic version of the questionnaire is provided with this notice as S1 File.

Regarding the measurement validity concerns, the corresponding author stated that the use of binary response options and the sequencing of items relating to support for FGM/C and associated beliefs were intended to facilitate completion in a busy clinical environment and to estimate the prevalence of commonly documented beliefs reported in large-scale international surveys. The corresponding author acknowledged that this design may introduce ambiguity as to whether respondents were reporting perceived community beliefs or personal endorsement, and that question sequencing may influence interpretation. They further stated that the inclusion of the statement “makes the practice safer” in the “medicalization” item was intended to measure the prevalence of a well‑documented misconception among healthcare providers rather than to normalize or endorse the practice and was balanced by the following two response options in the “medicalization” item.

The *PLOS Global Public Health* Editors consider that, notwithstanding prior validation of the original instrument, the concerns regarding measurement validity in the Egyptian context are not fully resolved, including the limited piloting sample, reliance on binary and highly loaded response options, and item sequencing issues.

## Study design and hypotheses

The consulted Editorial Board Member advised that the study’s hypotheses appear grounded in assumptions that may create risks of sociocultural and interpretive bias in framing determinants of FGM support and interpretive or confirmation bias in subgroup comparisons. The corresponding author stated that the hypotheses were informed by previous peer-reviewed literature, the Egyptian Demographic and Health Survey, and the national context of FGM/C practice and because the analyses were associative and ultimately did not support those expectations, the findings were not interpreted in a confirmatory manner. To minimize expectancy and priming biases, the questionnaire was self-administered, standardized across participants, and introduced with a neutral consent statement emphasizing voluntariness and anonymity. The corresponding author noted that social desirability bias is a limitation that may persist in surveys addressing sensitive topics. Finally, they stated that the hypotheses did not inform questionnaire design, data collection, or analytic procedures.

## Errors in reporting results

Errors in data reporting were identified and reported by the corresponding author post-publication, and further errors were identified in editorial follow-up. Specifically:

- In Table 2, some Yes/No totals were incorrectly matched with their questionnaire items.
- In Tables 3–5, the Yes/No responses to the item “Do you think that girls that have not undergone FGM should be discriminated” were reported with the coding incorrectly swapped (“Yes” response values reported as “No” and vice versa).
- In Table 4, the p-value for the item “It helps to maintain their virginity for their husband,” is incorrect; the correct value is approximately 0.06.
- In Table 5, the p-value for the item “As a health care provider, have you ever carried out FGM/C on a girl?” is incorrect; the correct value is approximately 0.053.

Corrected versions of Tables 2–5 are provided with this notice. The corresponding author stated that these corrections do not affect subsequent analyses or study conclusions.

- There are errors in the article text:
  ◦ In the Abstract, there is an error in the tenth and eleventh sentences. The correct sentences are: In the knowledge domain, the highest consequence identified was difficulty during delivery. In attitudes, 63% believed that FGM/C should continue, while 51% agreed that the HCPs have a role in eliminating FGM/C.
  ◦ In the Results section, there is an error in the third and fourth sentences of the second paragraph. The correct sentences are: In the knowledge domain in Table 2, the highest consequence identified was difficulty during delivery. In attitudes, 63% believed that FGM/C should continue, while 51% agreed that the HCPs have a role in eliminating FGM/C.
  ◦ In the Results section, there is an error in the first and second sentences of the fourth paragraph. The correct sentences are: As seen in Table 2, 60% of the respondents believed that FGM/C is a mandatory religious practice. Regarding the breakdown, as in Table 4, 73% of the Christian respondents agreed, while only 58% of the Muslim respondents agreed. Most questions did not result in a statistically significant association based on religion, except for the item “Do you think that the practice of FGM can ever be eliminated in Egypt?”.
  ◦ In the Discussion section, there is an error in the second sentence of the fourth paragraph. The correct sentence is: As shown in Table 2, 60% of the respondents believe it is a religious practice, while 58% believe it is a cultural practice.
  ◦ In the Discussion section, there is an error in the first sentence of the seventh paragraph. The correct sentence is: Concerning participants’ religion and reasons for supporting FGM/C, in the current study, approximately 73% of Christian HCP and 58% of Muslims supported the procedures, as in Table 4.
  ◦ In the Discussion section, there is an error in the second sentence of the eighth paragraph. The correct sentence is: The most commonly cited reasons for the performance of the procedure, according to women female participants, were: reduction of sexual feelings (61.3%), mandatory religious practice (61.3%), and deeply rooted cultural practice (61.3%), as compared to male participants who noted: reduction of sexual feelings (58%), mandatory religious practice (58%), good practice (58%) and reduction of the rate of prostitution (58%), as the most common reasons, as in Table 5.
  ◦ In the Discussion section, there is an error in the first sentence of the sixteenth paragraph. The correct sentence is: Concerning respondents’ attitudes toward the medicalization of the practice, approximately 51% of participants believed that HCP could play a role in eliminating the practice, and around 65% reported that this makes the practice safer, as in Table 2.

The corresponding author provided the corrected data and underlying records promptly upon request; however, the *PLOS Global Public Health* Editors note that the accumulated errors in data reporting raise concerns about the data management practices and reliability of the reported results.

In light of the above issues, the *PLOS Global Public Health* Editors issue this Expression of Concern.

The following additional issues were addressed in follow-up.

Concerns were raised about the presence of repeated numerical patterns in Tables 4 and 5. Upon follow-up, the corresponding author provided the underlying individual-level data for Tables 4 and 5 for editorial review and stated that the patterns result from the binary questionnaire design and fixed subgroup sizes, which sometimes produced identical 2x2 tables and therefore identical statistics.

The *PLOS Global Public Health* Editors consider the concerns raised about repeated numerical patterns to be resolved.

The study reports self-disclosure of support for, and participation in, female genital mutilation (FGM), which is illegal under Egyptian law and subject to criminal penalties. The combination of a bounded sample, workplace setting, purposive sampling, and admissions of illegal activity raises questions about whether confidentiality and participant protection were sufficiently robust. Upon follow-up, the corresponding author stated that the study ensured anonymity, voluntariness, and ethical oversight by using coded, non‑identifiable surveys, independent consent procedures, and these safeguards were approved by the institutional ethics committee.

**Table 2:** The overall results of the responses.

	Questions	Yes		No		Total
	n	%	n	%	n
**Reasons given by people to support FGM**	**Do you support performing the FGM procedure**	134	60.09	89	39.91	223
	**It is a mandatory religious practice**	134	60.09	89	39.91	223
	**It is a deeply rooted cultural practice**	130	58.3	93	41.7	223
	**It reduces sexual feelings**	134	60.09	89	39.91	223
	**It is a rite of passage for girls into womanhood**	129	57.85	94	42.15	223
	**It is a good practice**	133	59.64	90	40.36	223
	**It helps to maintain their virginity for their husband**	129	57.85	94	42.15	223
	**It reduces the rate of prostitution**	131	58.74	92	41.26	223
**Potential consequences of FGM**	**Transmission of infectious diseases**	90	40.36	133	59.64	223
	**Bleeding**	90	40.36	133	59.64	223
	**Health problems**	90	40.36	133	59.64	223
	**Difficulty during delivery**	92	41.26	131	58.74	223
	**Reduction of sexual feelings**	87	39.01	136	60.99	223
	**Affects the health and welfare of women and girls**	88	39.46	135	60.54	223
	**Difficult penetration during sex**	87	39.01	136	60.99	223
	**No consequences**	89	39.91	134	60.09	223
	**Has seen a girl with complications after FGM**	91	40.81	132	59.19	223
**Attitude towards FGM**	**Do you think that the practice of FGM should continue?**	141	63.23	82	36.77	223
	**Do you think that girls that have not undergone FGM should be discriminated?**	143	64.13	80	35.87	223
	**Do you think that the practice of FGM can ever be eliminated in Egypt?**	120	53.81	103	46.19	223
	**Do you think it is a good idea for men to be concerned on the debate on FGM?**	112	50.22	111	49.78	223
	**Do you think HCP workers have a role to play in eliminating FGM?**	114	51.12	109	48.88	223
	**Regarding medicalizing FGM, a) It makes the practice safer**	144	64.57	79	35.43	223
	**Regarding medicalizing FGM, b) It is a way of encouraging FGM**	144	64.57	79	35.43	223
	**Regarding medicalizing FGM c) It should be stopped at all levels**	95	42.6	128	57.4	223
**Practice towards FGM**	**Was FGM practiced in your family/household?**	102	45.74	121	54.26	223
	**Is FGM practiced in your family/household?**	108	48.43	115	51.57	223
	**If you have a daughter in the future, do you intend to circumcise her?**	138	61.88	85	38.12	223
	**As a health care provider, have you ever carried out FGM/C on a girl?**	9	4.04	214	95.96	223

**Table 3:** Comparison based on the place of residence of the respondents and the p-value for the association based on the place of residence.

			Residence					
			Urban (n = 111)		Rural (n = 112)			
			Count	Column N %	Count	Column N %	X^2^	p value
**Reasons given by people to support FGM**	Do you support performing the FGM procedure	Yes	32	28.80%	102	91.10%	90.059	<0.001
		No	79	71.20%	10	8.90%		
	It is a mandatory religious practice	Yes	32	28.80%	102	91.10%	90.059	<0.001
		No	79	71.20%	10	8.90%		
	It is a deeply rooted cultural practice	Yes	29	26.10%	101	90.20%	94.079	<0.001
		No	82	73.90%	11	9.80%		
	It reduces sexual feelings	Yes	32	28.80%	102	91.10%	90.059	<0.001
		No	79	71.20%	10	8.90%		
	It is a rite of passage for girls into womanhood	Yes	29	26.10%	100	89.30%	91.203	<0.001
		No	82	73.90%	12	10.70%		
	It is a good practice	Yes	32	28.80%	101	90.20%	87.172	<0.001
		No	79	71.20%	11	9.80%		
	It helps to maintain their virginity for their husband	Yes	29	26.10%	100	89.30%	91.203	<0.001
		No	82	73.90%	12	10.70%		
	It reduces the rate of prostitution	Yes	32	28.80%	99	88.40%	81.612	<0.001
		No	79	71.20%	13	11.60%		
**Potential consquences of FGM**	Transmission of infectious diseases	Yes	70	63.10%	20	17.90%	47.331	<0.001
		No	41	36.90%	92	82.10%		
	Bleeding	Yes	70	63.10%	20	17.90%	47.331	<0.001
		No	41	36.90%	92	82.10%		
	Health problems	Yes	70	63.10%	20	17.90%	47.331	<0.001
		No	41	36.90%	92	82.10%		
	Difficulty during delivery	Yes	71	64.00%	21	18.80%	47.025	<0.001
		No	40	36.00%	91	81.30%		
	Reduction of sexual feelings	Yes	67	60.40%	20	17.90%	42.328	<0.001
		No	44	39.60%	92	82.10%		
	Affects the health and welfare of women and girls	Yes	68	61.30%	20	17.90%	43.963	<0.001
		No	43	38.70%	92	82.10%		
	Difficult penetration during sex	Yes	67	60.40%	20	17.90%	42.328	<0.001
		No	44	39.60%	92	82.10%		
	No consequences	Yes	68	61.30%	21	18.80%	42.011	<0.001
		No	43	38.70%	91	81.30%		
	Has seen a girl with complications after FGM	Yes	71	64.00%	20	17.90%	49.064	<0.001
		No	40	36.00%	92	82.10%		
**Attitude towards FGM**	Do you think that the practice of FGM should continue?	Yes	46	41.40%	95	84.80%	45.122	<0.001
		No	65	58.60%	17	15.20%		
	Do you think that girls that have not undergone FGM should be discriminated?	Yes	48	43.20%	95	84.80%	41.894	<0.001
		No	63	56.80%	17	15.20%		
	Do you think that the practice of FGM can ever be eliminated in Egypt?	Yes	57	51.40%	63	56.30%	0.538	0.46
		No	54	48.60%	49	43.80%		
	Do you think it is a good idea for men to be concerned on the debate on FGM?	Yes	61	55.00%	51	45.50%	1.979	0.16
		No	50	45.00%	61	54.50%		
	Do you think HCP workers have a role to play in eliminating FGM?	Yes	60	54.10%	54	48.20%	0.761	0.38
		No	51	45.90%	58	51.80%		
	a) It makes the practice safer	Yes	71	64.00%	73	65.20%	0.036	0.85
		No	40	36.00%	39	34.80%		
	b) It is a way of encouraging FGM	Yes	71	64.00%	73	65.20%	0.036	0.85
		No	40	36.00%	39	34.80%		
	c) It should be stopped at all levels	Yes	74	66.70%	21	18.80%	52.346	<0.001
		No	37	33.30%	91	81.30%		
**Practice towards FGM**	Was FGM practiced in your family/household?	Yes	45	40.50%	57	50.90%	2.407	0.121
		No	66	59.50%	55	49.10%		
	Is FGM practiced in your family/household?	Yes	53	47.70%	55	49.10%	0.041	0.84
		No	58	52.30%	57	50.90%		
	If you have a daughter in the future, do you intend to circumcise her?	Yes	49	44.10%	89	79.50%	29.484	<0.001
		No	62	55.90%	23	20.50%		
	As a health care provider, have you ever carried out FGM/C on a girl?	Yes	4	3.60%	5	4.50%	0.107	0.74
		No	107	96.40%	107	95.50%		

**Table 4:** Comparison based on the religion of the respondents and the p-value for the association based on the religion reported.

			Religion					
			Muslim (n = 190)		Christian (n = 33)			
			Count	Column N %	Count	Column N %	X^2^	p value
**Reasons given by people to support FGM**	Do you support performing the FGM procedure	Yes	110	57.90%	24	72.70%	2.579	0.11
		No	80	42.10%	9	27.30%		
	It is a mandatory religious practice	Yes	110	57.90%	24	72.70%	2.579	0.11
		No	80	42.10%	9	27.30%		
	It is a deeply rooted cultural practice	Yes	106	55.80%	24	72.70%	3.318	0.07
		No	84	44.20%	9	27.30%		
	It reduces sexual feelings	Yes	110	57.90%	24	72.70%	2.579	0.11
		No	80	42.10%	9	27.30%		
	It is a rite of passage for girls into womanhood	Yes	105	55.30%	24	72.70%	3.517	0.06
		No	85	44.70%	9	27.30%		
	It is a good practice	Yes	109	57.40%	24	72.70%	2.755	0.09
		No	81	42.60%	9	27.30%		
	It helps to maintain their virginity for their husband	Yes	105	55.30%	24	72.70%	3.517	0.06
		No	85	44.70%	9	27.30%		
	It reduces the rate of prostitution	Yes	107	56.30%	24	72.70%	3.125	0.08
		No	83	43.70%	9	27.30%		
**Potential consequences of FGM**	Transmission of infectious diseases	Yes	75	39.50%	15	45.50%	0.418	0.52
		No	115	60.50%	18	54.50%		
	Bleeding	Yes	75	39.50%	15	45.50%	0.418	0.52
		No	115	60.50%	18	54.50%		
	Health problems	Yes	75	39.50%	15	45.50%	0.418	0.52
		No	115	60.50%	18	54.50%		
	Difficulty during delivery	Yes	76	40.00%	16	48.50%	0.835	0.36
		No	114	60.00%	17	51.50%		
	Reduction of sexual feelings	Yes	71	37.40%	16	48.50%	1.460	0.23
		No	119	62.60%	17	51.50%		
	Affects the health and welfare of women and girls	Yes	74	38.90%	14	42.40%	0.142	0.71
		No	116	61.10%	19	57.60%		
	Difficult penetration during sex	Yes	73	38.40%	14	42.40%	0.189	0.66
		No	117	61.60%	19	57.60%		
	No consequences	Yes	73	38.40%	16	48.50%	1.187	0.28
		No	117	61.60%	17	51.50%		
	Has seen a girl with complications after FGM	Yes	76	40.00%	15	45.50%	0.346	0.57
		No	114	60.00%	18	54.50%		
**Attitude towards FGM**	Do you think that the practice of FGM should continue?	Yes	118	62.10%	23	69.70%	0.697	0.4
		No	72	37.90%	10	30.30%		
	Do you think that girls that have not undergone FGM should be discriminated?	Yes	120	63.20%	23	69.70%	0.523	0.47
		No	70	36.80%	10	30.30%		
	Do you think that the practice of FGM can ever be eliminated in Egypt?	Yes	108	56.80%	12	36.40%	4.744	0.03
		No	82	43.20%	21	63.60%		
	Do you think it is a good idea for men to be concerned on the debate on FGM?	Yes	95	50.00%	17	51.50%	0.026	0.87
		No	95	50.00%	16	48.50%		
	Do you think HCP workers have a role to play in eliminating FGM?	Yes	97	51.10%	17	51.50%	0.002	0.96
		No	93	48.90%	16	48.50%		
	a) It makes the practice safer	Yes	122	64.20%	22	66.70%	0.074	0.79
		No	68	35.80%	11	33.30%		
	b) It is a way of encouraging FGM	Yes	122	64.20%	22	66.70%	0.074	0.79
		No	68	35.80%	11	33.30%		
	c) It should be stopped at all levels	Yes	84	44.20%	11	33.30%	1.360	0.24
		No	106	55.80%	22	66.70%		
**Practice towards FGM**	Was FGM practiced in your family/household?	Yes	84	44.20%	18	54.50%	1.210	0.27
		No	106	55.80%	15	45.50%		
	Is FGM practiced in your family/household?	Yes	90	47.40%	18	54.50%	0.580	0.45
		No	100	52.60%	15	45.50%		
	If you have a daughter in the future, do you intend to circumcise her?	Yes	117	61.60%	21	63.60%	0.050	0.82
		No	73	38.40%	12	36.40%		
	As a health care provider, have you ever carried out FGM/C on a girl?	Yes	8	4.20%	1	3.00%	0.101	0.75
		No	182	95.80%	32	97.00%		

**Table 5:** Comparison based on the gender of the respondents and the p-value for the association based on gender.

			Gender					
			Male (n = 81)		Female (n = 142)			
			Count	Column N %	Count	Column N %	X^2^	p value
**Reasons given by people to support FGM**	Do you support performing the FGM procedure	Yes	47	58.00%	87	61.30%	0.226	0.63
		No	34	42.00%	55	38.70%		
	It is a mandatory religious practice	Yes	47	58.00%	87	61.30%	0.226	0.63
		No	34	42.00%	55	38.70%		
	It is a deeply rooted cultural practice	Yes	43	53.10%	87	61.30%	1.42	0.23
		No	38	46.90%	55	38.70%		
	It reduces sexual feelings	Yes	47	58.00%	87	61.30%	0.2226	0.63
		No	34	42.00%	55	38.70%		
	It is a rite of passage for girls into womanhood	Yes	44	54.30%	85	59.90%	0.649	0.42
		No	37	45.70%	57	40.10%		
	It is a good practice	Yes	47	58.00%	86	60.60%	0.138	0.71
		No	34	42.00%	56	39.40%		
	It helps to maintain their virginity for their husband	Yes	43	53.10%	86	60.60%	1.183	0.28
		No	38	46.90%	56	39.40%		
	It reduces the rate of prostitution	Yes	47	58.00%	84	59.20%	0.027	0.87
		No	34	42.00%	58	40.80%		
**Potential consequences of FGM**	Transmission of infectious diseases	Yes	44	54.30%	46	32.40%	10.302	0.001
		No	37	45.70%	96	67.60%		
	Bleeding	Yes	44	54.30%	46	32.40%	10.302	0.001
		No	37	45.70%	96	67.60%		
	Health problems	Yes	44	54.30%	46	32.40%	10.302	0.001
		No	37	45.70%	96	67.60%		
	Difficulty during delivery	Yes	45	55.60%	47	33.10%	10.733	0.001
		No	36	44.40%	95	66.90%		
	Reduction of sexual feelings	Yes	41	50.60%	46	32.40%	7.199	0.007
		No	40	49.40%	96	67.60%		
	Affects the health and welfare of women and girls	Yes	42	51.90%	46	32.40%	8.174	0.004
		No	39	48.10%	96	67.60%		
	Difficult penetration during sex	Yes	42	51.90%	45	31.70%	8.812	0.003
		No	39	48.10%	97	68.30%		
	No consequences	Yes	41	50.60%	48	33.80%	6.081	0.014
		No	40	49.40%	94	66.20%		
	Has seen a girl with complications after FGM	Yes	44	54.30%	47	33.10%	9.617	0.002
		No	37	45.70%	95	66.90%		
**Attitude towards FGM**	Do you think that the practice of FGM should continue?	Yes	50	61.70%	91	64.10%	0.123	0.73
		No	31	38.30%	51	35.90%		
	Do you think that girls that have not undergone FGM should be discriminated?	Yes	52	64.20%	91	64.10%	0	0.97
		No	29	35.80%	51	35.90%		
	Do you think that the practice of FGM can ever be eliminated in Egypt?	Yes	38	46.90%	82	57.70%	2.435	0.12
		No	43	53.10%	60	42.30%		
	Do you think it is a good idea for men to be concerned on the debate on FGM?	Yes	45	55.60%	67	47.20%	1.446	0.23
		No	36	44.40%	75	52.80%		
	Do you think HCP workers have a role to play in eliminating FGM?	Yes	43	53.10%	71	50.00%	0.197	0.66
		No	38	46.90%	71	50.00%		
	a) It makes the practice safer	Yes	56	69.10%	88	62.00%	1.157	0.28
		No	25	30.90%	54	38.00%		
	b) It is a way of encouraging FGM	Yes	56	69.10%	88	62.00%	1.157	0.28
		No	25	30.90%	54	38.00%		
	c) It should be stopped at all levels	Yes	48	59.30%	47	33.10%	14.436	<0.001
		No	33	40.70%	95	66.90%		
**Practice towards FGM**	Was FGM practiced in your family/household?	Yes	42	51.90%	60	42.30%	1.915	0.17
		No	39	48.10%	82	57.70%		
	Is FGM practiced in your family/household?	Yes	35	43.20%	73	51.40%	1.388	0.24
		No	46	56.80%	69	48.60%		
	If you have a daughter in the future, do you intend to circumcise her?	Yes	38	46.90%	100	70.40%	12.085	0.001
		No	43	53.10%	42	29.60%		
	As a health care provider, have you ever carried out FGM/C on a girl?	Yes	6	7.40%	3	2.10%	3.733	0.053
		No	75	92.60%	139	97.90%		

## Supporting information

S1 File FGM questionnaire Arabic. (DOCX)
