# Supplementary material for: Knowledge, attitudes, and practices of Female Genital Mutilation / Cutting among healthcare providers in two public hospitals in Egypt: A cross-sectional study
Source: PLOS Glob Public Health. 2023 Dec 29;3(12):e0002724. doi: 10.1371/journal.pgph.0002724 (PMC10756516; doi:10.1371/journal.pgph.0002724)
Supplement: S1 Questionnaire — (DOCX) [file pgph.0002724.s001.docx]

**FGM/C – Questionnaire**

**Informed Consent Statement:** “Your participation in this survey is completely voluntary. All the information you provide for the study will be kept completely confidential. We record your responses, but the questionnaire will not have your name on it, and your responses to our questions are identified only by a number, never by name. We hope that this survey will be considered a baseline assessment that will guide us in improving the knowledge and attitude of HCPs towards FGM/C, which is considered an important step in combating it. The survey will take about 10-15 minutes.”

**Instructions**: This survey asks for your opinions about FGM/C and will take about 10 to 15 minutes to complete. If you do not wish to answer a question, or if a question does not apply to you, you may leave your answer blank.

***The information derived from this questionnaire to be used in this study are strictly confidential and are used for scientific purposes only.***

#### Part I: Socio-demographic background

**1) Sex:**

1. Male.
2. Female.

1. **Age (in Years): _______________.**

1. **Residence:**

1-Urban

2-Rural

1. **Religion:**

1. Muslim.
2. Christian.
3. Other: Mention ______________________.

**5) Education level:**

1-Bachelor degree

2-Master degree

3-PHD / MD

1. Diploma
2. Fellowship

#### Part II: Occupational background

**6) Select ONE answer regarding your health facility:**

1. Primary Healthcare Unit.
2. Secondary hospital.
3. Tertiary/teaching hospital.
4. Other (Specify_____________)

**7) Select ONE answer regarding your health facility:**

1. Governmental (Ministry of Health).
2. Governmental (Ministry of Education).
3. Non-Governmental Organization.
4. Private.
5. Other (Specify_______________

**8) Select ONE answer regarding your work area:**

1. Obstetrics and Gynecology.
2. Medicine (non-surgical).
3. Surgery.
4. Pharmacy.
5. Emergency department.
6. Anesthesiology.
7. Intensive care unit
8. Physiotherapy/rehabilitation center
9. Other: Mention ______________

**9) Select ONE answer regarding your job title:**

1. Resident physician/ physician in training.
2. Registered nurse.
3. Physical Therapist.
4. Head nurse.
5. Pharmacist.
6. Attending/ Staff physician.
7. Other (Please specify-----------------).

**10) How long have you worked in this field?**

1. Less than 1 year. 2. 1 to 5 years

3. 6 to 10 years. 4. 11 to 15 years

5. 16 to 20 years. 6. 21 years or more

#### Part III: Knowledge of FGM/C

**11) Do you support performing the FGM/C procedure? Yes No**

| **A) What do you think are the reasons given by people who support FGM/C** | | | |
| --- | --- | --- | --- |
| **No.** | **Questions** | **Responses** | |
|  |  | **Yes** | **No** |
| **12** | It is a mandatory religious practice |  |  |
| **13** | It is a deeply rooted cultural practice. |  |  |
| **14** | It reduces sexual feelings. |  |  |
| **15** | It is a rite of passage for girls into womanhood. |  |  |
| **16** | It is a good practice. |  |  |
| **17** | It helps to maintain their virginity for their husband. |  |  |
| **18** | It reduces the rate of prostitution. |  |  |

| **B) Are you aware of the potential consequences of FGM/C** | | | |
| --- | --- | --- | --- |
| **No.** | **Questions** | **Responses** | |
|  |  | **Yes** | **No** |
| **19** | Health problems |  |  |
| **20** | Bleeding |  |  |
| **21** | Transmission of infectious diseases |  |  |
| **22** | Difficulty during delivery |  |  |
| **23** | Reduction of sexual feelings |  |  |
| **24** | It affects the health and welfare of women and girls |  |  |
| **25** | Difficult penetration during sex |  |  |
| **26** | No consequences |  |  |
| **27** | Has seen a girl with complications after FGM/C |  |  |

Part V: Attitude towards FGM/C:

| **No.** | **Questions** | **Responses** | |
| --- | --- | --- | --- |
|  |  | **Yes** | **No** |
| **28** | Do you think that the practice of FGM/C should continue? |  |  |
| **29** | Do you think girls who have not undergone FGM/C should be discriminated against? |  |  |
| **30** | Do you think that the practice of FGM/C can ever be eliminated in Egypt? |  |  |
| **31** | Do you think it is a good idea for men to be concerned about the debate on FGM/C? |  |  |
| **32** | Do you think HCP workers have a role to play in eliminating FGM/C? |  |  |
| **33** | What do you think of ‘medicalizing’ FGM/C? |  |  |
|  | a) It makes the practice safer |  |  |
|  | b) It is a way of encouraging FGM/C |  |  |
|  | c) It should be stopped |  |  |

Part VI: Practice of FGM/C:

| **No.** | **Questions** | **Responses** | |
| --- | --- | --- | --- |
|  |  | **Yes** | **No** |
| **34** | Was FGM/C practiced in your family/household? |  |  |
| **35** | Is FGM/C still being practiced in your family/household? |  |  |
| **36** | If you have a daughter in the future, do you intend to circumcise her? |  |  |
| **37** | As a health care provider, have you ever carried out FGM/C on a girl? |  |  |

**THANK YOU SO MUCH FOR COMPLETING THIS SURVEY**
