# Supplementary material for: Expression of Concern: Knowledge, attitudes, and practices of Female Genital Mutilation / Cutting among healthcare providers in two public hospitals in Egypt: A cross-sectional study
Source: PLOS Glob Public Health. 2026 Aug 26;6(8):e0007200. doi: 10.1371/journal.pgph.0007200 (PMC13514045; doi:10.1371/journal.pgph.0007200)
Supplement: Supplementary file 1 [file pgph.0007200.s001.docx]

**استبيان عن ختان الإناث**

***المعلومات المستقاة من هذا الاستبيان سيتم استخدامها في دراسة*** عن آرائكم و معرفتكم بختان الإناث، وسوف يستغرق حوالي 5 إلى 10 دقائق لإكماله.

**الموافقة المستنيرة:** "مشاركتكم في هذا الاستطلاع طوعية تماما. سيتم الاحتفاظ بسرية جميع المعلومات التي تقدمها للدراسة. نحن نسجل ردودكم، ولكن الاستبيان لن يكون عليه اسمك، ويتم تحديد ردودكم على أسئلتنا فقط من خلال الرقم، ليس بالاسم. ونأمل أن يعتبر هذا المسح تقييماً أساسياً يرشدنا في تحسين معرفة مقدمي الخدمة الصحية عن ختان الإناث.

**تعليمات:** إذا كنت لا ترغب في الإجابة عن سؤال، أو إذا وجدت سؤال لا ينطبق عليك، قد تترك الإجابة فارغة.

***جميع البيانات ستعامل بسرية تامة وتستخدم لأغراض علمية فقط.***

**الجزء الأول: الخلفية الاجتماعية – الديمغرافية**

**1) الجنس:**

1- ذكر. 2- أنثى.

**2) العمر بالسنوات:** ______________________

**3) مكان الإقامة:**

1- الحضر	2- الريف

**4) الديانة:**

1- مسلم	2- مسيحي

3- غير ذلك (اذكر):______________________

**5) مستوى التعليم:**

1- درجة البكالوريوس	2- درجة الماجستير

3- درجة الدكتوراه	4- درجة دبلوم

5- درجة الزمالة

**الجزء الثاني: الخلفية المهنية**

**6) حدد إجابة واحدة فيما يتعلق بالمرفق الصحي الخاص بك**:

1- وحدة الرعاية الصحية الأولية. 2- مستشفى ثانوي.

3- مستشفى من مستوى ثالث / تعليمي. 4- أخرى: حدد (_____________)

**7) حدد إجابة واحدة فيما يتعلق بالمرفق الصحي الخاص بك**:

1- حكومي (وزارة الصحة). 2- حكومي (وزارة التعليم العالي).

3- المنظمات غير الحكومية. 4- خاص.

5- أخرى: حدد (_____________)

**8) حدد إجابة واحدة فيما يتعلق بمنطقة العمل الخاصة بك**:

1. النساء و التوليد. 2- الباطنة (غير الجراحية).

3. الجراحة. 4- الصيدلة

5. قسم الطوارئ. 6- التخدير.

7. وحدة العناية المركزة	8- العلاج الطبيعي / مركز إعادة التأهيل

9. أخرى: حدد (_____________)

**9) حدد إجابة واحدة فيما يتعلق بالتوصيف الوظيفي:**

1. طبيب مقيم / طبيب في التدريب. 2- ممرضة مسجلة.

3. العلاج الطبيعي. 4- رئيس تمريض

5- صيدلي. 6- طبيب مشرف

7- أخرى (حدد-----------------).

**10) منذ متى و أنت تعمل في هذا المجال؟**

1. أقل من 1 سنة. 2. 1 إلى 5 سنوات

3. 6 إلى 10 سنوات. 4- 11 إلى 15 سنة

5. 16 إلى 20 سنة. 6. 21 سنة أو أكثر

**الجزء الثالث: المعرفة بختان الإناث**

| **الأسباب التي قدمها الأشخاص الذين يؤيدون ختان الإناث** | | | |
| --- | --- | --- | --- |
| **الرقم** | **السؤال** | **نعم** | **لا** |
| **11** | إنها ممارسة دينية إلزامية |  |  |
| **12** | إنها ممارسة ثقافية عميقة الجذور |  |  |
| **13** | أنه يقلل من المشاعر الجنسية |  |  |
| **14** | إنها طقوس أساسية لمرور الفتيات إلى الأنوثة |  |  |
| **15** | إنها ممارسة جيدة |  |  |
| **16** | يساعد على الحفاظ على عذريتهم لزوجهم |  |  |
| **17** | يقلل الختان من معدل البغاء |  |  |
| **18** | لا ينتهك الختان حقوق الإنسان |  |  |

| **الإجابات المبلغ عنها من ممارسي الخدمات الصحية حول العواقب الصحية لختان الإناث** | | | |
| --- | --- | --- | --- |
| **الرقم** | **السؤال** | **نعم** | **لا** |
| **19** | انتقال الأمراض المعدية |  |  |
| **20** | النزيف |  |  |
| **21** | المشاكل الصحية |  |  |
| **22** | صعوبة أثناء الولادة |  |  |
| **23** | الحد من المشاعر الجنسية |  |  |
| **24** | يؤثر على صحة النساء والفتيات |  |  |
| **25** | اختراق أصعب أثناء ممارسة الجماع |  |  |
| **26** | لا يوجد عواقب |  |  |
| **27** | شهدت فتاة مع مضاعفات بعد ختان الإناث |  |  |
| **28** | يعتبر ختان الإناث ممارسة دينية إلزامية |  |  |

| **الموقف تجاه ختان الإناث** | | | |
| --- | --- | --- | --- |
| **الرقم** | **السؤال** | **نعم** | **لا** |
| **29** | هل تعتقد أن ممارسة ختان الإناث يجب أن تستمر؟ |  |  |
| **30** | هل تعتقد أن الفتيات اللواتي لم يخضعن للختان يجب أن يتعرضن للتمييز؟ |  |  |
| **31** | هل تعتقد أن ممارسة ختان الإناث يمكن القضاء عليها في مصر؟ |  |  |
| **32** | هل تعتقد أنها فكرة جيدة للرجال أن يهتموا بالنقاش حول ختان الإناث؟ |  |  |
| **33** | هل تعتقد أن العاملين في المجال الصحي لهم دور في القضاء على ختان الإناث؟ |  |  |
| **34** | ما رأيك في "إضفاء الطابع الطبي" على ختان الإناث؟ |  |  |
|  | أ) يجعل الممارسة أكثر أمانا |  |  |
|  | ب) هي طريقة لتشجيع ختان الإناث |  |  |
|  | ج) ينبغي وقفها على جميع المستويات |  |  |

| **الممارسات الخاصة بختان الإناث** | | | |
| --- | --- | --- | --- |
| **الرقم** | **السؤال** | **نعم** | **لا** |
| **35** | هل تم ممارسة ختان الإناث في عائلتك/أسرتك؟ |  |  |
| **36** | هل يتم ممارسة ختان الإناث في عائلتك/أسرتك؟ |  |  |
| **37** | إذا كان لديك ابنة في المستقبل، هل تنوي ختانها؟ |  |  |
| **38** | كمقدم رعاية صحية، هل سبق لك أن قمت بختان أي فتاة؟ |  |  |

شكرا جزيلا لمشاركتك في هذه الدراسة

نرجو ان ترسل هذا الإستبيان لأحد زملائك بالعمل
